# Commercial Postural Devices: A Review

**DOI:** 10.3390/s19235128

**Published:** 2019-11-23

**Authors:** Nicole Kah Mun Yoong, Jordan Perring, Ralph Jasper Mobbs

**Affiliations:** 1 Faculty of Medicine, University of New South Wales, Sydney 2052, Australia; jordanperringjp@gmail.com (J.P.); r.mobbs@unsw.edu.au (R.J.M.); 2 NeuroSpine Surgery Research Group (NSURG), Sydney 2052, Australia; 3 Department of Neurosurgery, Prince of Wales Hospital, Sydney 2052, Australia

**Keywords:** postural analysis, wearable technology, commercial devices, spinal posture

## Abstract

Wearables are devices worn on the human body and are able to measure various health parameters, such as physical activity, energy expenditure and gait. With the advancement of technology, the general population are now spending more hours craning our necks and slouching over smartphones, tablets and computers, et cetera. Bodily posture is representative of physical and mental health. Poor posture can lead to spinal complications and the same can be said vice versa. As the standard of living increases, there is an increase in consumerism and the expectation to maintain such a lifestyle even in the aging population. Therefore, many are able to afford small luxuries in life, such as a piece of technology that could potentially improve their health in the long run. Wearable technology is a promising alternative to laboratory systems for movement and posture analysis. This article reviews commercial wearable devices with a focus on postural analysis. The clinical applicability of posture wearables, particularly in preventing, monitoring and treating spinal and musculoskeletal conditions, along with other purposes in healthcare, will be discussed.

## 1. Introduction

The word posture is of French origin—posteure—which means the relative disposition of parts; in particular, the carriage and position of limbs or the body as a whole, indicating a certain feeling, pose, attitude or quality [1]. Evolutionary framework depicts the upright posture as the quality that differentiates the earliest human beings from its predecessors. The recent discovery of *Ardipithecus ramidus* as our ancient ancestor was defined by its upright gait. Its skeleton revealed an intermediate form of upright walking which is considered a hallmark of humans. It showed signs of bipedalism, upright walking on both feet and standing rigidly upright. Studies suggest that this plumb line upright posture was due to its habitat in woodlands with high grass, where one had to be able to look over the grass to see prey [2]. This upright posture can be defined as bipedalism, and although a variety of primates and other mammals perform this, human ancestors adopted this as their exclusive form of locomotion, and thenceforth evolved and adapted around it. Ancient culture also describes upright posture as the one defining qualities that makes us humans. For instance, Aristotle and Plato describe man as the only being standing upright. An Ancient Greek word for men, Anthropos, means the animal that looks upwards and considers his god [1].

Posture is a flexible concept between static, mechanics, gait and activities. While the position of the body is static at rest and during gait, the way the body moves in space and time during activities consists of both static and mechanical movement [1]. Postural control is the positional control of the whole body in space for balance and orientation, whereas postural orientation is the maintenance of appropriate relationships between body segments [3]. A neutral posture is when the head and upper trunk are at zero degrees to the vertebral column. Subsequent deviations from this neutral posture correlate to poor posture. Poor posture is linked to accelerated intervertebral disc (IVD) degeneration as well as damage and misalignment of vertebrae, resulting in nerve root compression [4]. This nerve root compression and impingement may lead to radicular symptoms, such as sensorimotor deficits and pain in the affected regions. Although poor posture can lead to complications involving the spine and its surrounding structures, the same can be said vice versa. Postural slump may represent fatigue, and poor muscular tone and mental state. To a keen eye, direct visual observation of one’s posture could reveal signs of deep-seated disability, pain, low quality of life and even mental concomitants of poor posture [5]. Therefore, body posture can act as an indicator of health itself.

Wearable devices are defined as electronic devices or computers that can be worn [4]. With advances in such technology, the notion of utilizing wearables in healthcare is slowly emerging with the concept of a ’quantified self’. It is hypothesized that these devices can educate and motivate individuals to adopt healthy lifestyles via recording and reporting information such as postural control and physical activity [6]. Wearable devices in the field of medicine first emerged in the commercial market with cochlear implants, whereby an artificial cochlear capable of transmitting nerve signals was proven to be successful in overcoming hearing loss. The use of wearables in the fitness industry is not a foreign concept, with one in six consumers in the United States using wearable technology such as smartwatches and fitness bands [7]. The outlook of wearables is optimistic with 142 million units shipped worldwide in the year 2019 and an estimated 260 million unit sales by the year 2023 [8]. The use of wearable devices to delineate spinal pathologies and as an indicator of health status in a free-living scenario are up and coming avenues for healthcare.

There are various mechanical and neurological pathologies which result in disorders of posture. Common examples would be lumbar spinal stenosis with a stooped posture, myelopathy with significant postural instability and Parkinson’s Disease with a variety of postural changes in ambulation. There are, however, various inadequacies in the literature linking an objective assessment with detection of various neurological and musculoskeletal conditions, in addition to an attempt to correct various postural issues with the use of wearable devices. In light of the potential of wearable devices in delineating spinal pathologies, the aim of this paper was to review the available postural devices on the commercial market. The principles of these wearable postural devices, including their components, movement classifications and sensor placements, will be explored. Furthermore, the clinical applicability of these devices, challenges with their employment and future steps in integrating posture wearables in daily life will be discussed.

## 2. Principles of Wearable Devices in Posture Analysis

Microelectromechanical systems (MEMS), which include inertial sensors such as accelerometers, gyroscopes and magnetometers are utilized in wearables. The most common underlying technology in wearables is the inertial measurement units (IMU), which typically consists of accelerometers and gyroscopes, and may include magnetometers. Components featured in IMUs are summarized in Table 1 [4].

### 2.1. Accelerometers

Accelerometers measure acceleration in different orientations so three-dimensional spatial movements be appreciated with the development of triaxial accelerometers from the previous uniaxial models. Roll and pitch angles can be obtained from the gravity components measured with an accelerometer in static state [9]. Raw accelerometry data can also be extracted to provide information on intensity of activities, posture, postural transitions and gait patterns [10]. Accelerometers can respond to gravity or constant acceleration—tilt sensing; hence, human postures such as upright and lying can be discriminated with the magnitude of acceleration signals from a single accelerometer at the waist and torso [11]. A single accelerometer is unable to accurately differentiate between standing and sitting as they are in upright positions [12]. This can be overcome with the use of multiple accelerometers to observe various orientations of body segments. For instance, standing and sitting postures can be differentiated from static activities by attaching two accelerometers, one to the torso and one to the thigh [13]. However, postural transitions from siting to standing can be recognized with a single accelerometer attached at the waist based on patterns of vertical acceleration [11].

### 2.2. Gyroscope

The gyroscope is a type of force sensor which detects angular motion about one or two axes [11]. The use of a gyroscope is vital as it can measure and maintain axial rotation as well as provide rotational and orientational information. Gyroscopes can be used to further expand the applicability of an accelerometer and magnetometer in orientation determination from a static to dynamic state [9]. Attached at the chest, a gyroscope can measure trunk-tilt variation due to sit-to-stand postural transitions [14]. Although gyroscope data alone is not as accurate as accelerometer data in providing body positional information, the combination of a gyroscope and accelerometer can achieve an average 95% accuracy [15]. However, there is a margin of error while using a single-point gyroscope due to the lack of a reference point and its inability to be reset to an initial state.

### 2.3. Magnetometers

The error in using a single-point gyroscope may be reduced with the use of magnetometers, which calibrate the device to the Earth’s magnetic field. The combination of an accelerometer and magnetometer known as a digital compass can determine orientation in a static state via measurements of time-dependent gravity and the Earth’s magnetic field vectors [9]. The use of digital compasses can avoid the drift and integration induced errors present in gyroscopes by providing an external reference to limit this drift [16]. Nonetheless, interference from other metal sources can affect the reading of magnetometers [17]. Therefore, modern wearable devices generally consist of a combination of all three monitors in all three axes; i.e., pitch (x-axis), roll (y-axis) and yaw (z-axis) [18].

### 2.4. Inertial Measurement Units

Inertial measurement units in the form of a combination of an accelerometer, gyroscope and magnetometer allow for the provision of redundant position and orientation data. IMUs allow measurement of rotational angle about a joint by identifying the main axis of motion. It is assumed that two segments are connected by a joint with one rotational degree of freedom (DoF). Commercial IMUs have developed towards multiple DoFs for better accuracy, as summarized in Table 2 [4].

### 2.5. Movement Classification

Movement classification can be achieved via statistical classification or can be threshold based. Such statistical schemes utilize supervised machine learning, which links features of a movement to possible movement states in terms of the possibility of the observation. Some examples of machine learning techniques include artificial neural networks, Gaussian mixture models (GMMs) and fuzzy logic [10]. In GMM, the likelihood function is not of a typical Gaussian distribution. An expectation-maximization algorithm is used to determine probability of activities. Threshold-based movement classification uses a hierarchical algorithm structure such as a decision tree model to differentiate activity states [11]. Movement detection and classification can be performed automatically. Mathie et al. [19] presented a classification framework of a hierarchical binary tree that can classify falling, walking and postural transitions using data from a wearable triaxial accelerometer. The individual algorithms in this model are also modifiable for different purposes.

### 2.6. Sensor Placement

Sensor placement of wearable devices comprises both the locations of these sensors on an individual and methods of attachment [20]. Sensor type and placement are vital in the recognition accuracy of body motions in various situations. For instance, accelerometers are better at identifying standing and sitting activities, whereas gyroscopes are better in recognizing descending and stair climbing motions. A ‘wearability map’ can be used to identify common areas for sensor placement; namely, the wrist, waist, sternum, lower back or foot. Waist-worn sensors are adopted in many studies as they are close to the body’s center of mass and because the torso occupies the majority of the mass of a human body, so sensor placement here can better represent most motions [20]. Waist-level devices are easily attached and detached from a belt; thus, minimizing discomfort and limitations in carrying out activities of daily living. Furthermore, the torso has greater weight-bearing ability when carrying wearables from an ergonomic perspective. Waist-worn accelerometers have been able to classify a range of basic daily activities such as walking, posture and activity transitions.

Commonly, motion sensors are attached to the human body either directly to the skin or indirectly via straps, belts, wristbands, clothing or other accessories. Integration of wearable devices into the clothing itself, termed smartfabric, is also possible. Nonetheless, these sensors have to be fitted and attached securely to the body to prevent relative motion between the sensor and body segments. Vibration and displacement of the wearable device due to unsecured or loose attachment can lead to reduced sensing accuracy and the production of extraneous signal artifacts [11].

## 3. Validity and Reliability of Wearable Devices for Postural Analysis

With the commercialization of wearable health technology, there is a concern regarding the validity and reliability of such devices, as many manufacturers either do not or only partially provide empirical evidence of product effectiveness. Furthermore, the apparent lack of wearable posture devices on the market as opposed to those for monitoring physical activity limits their availability for large-scale trials. Some commercial postural devices include LumoLift, LumoBack and Upright Go. However, the company behind Lumo declared bankruptcy in 2018. Although its products may still be available, users will not be able to receive any technical support. This further limits any possible support which can be given to researchers in this field. LumoLift and Upright Go are attached onto the wearer’s body either with a clasp or adhesive, providing a haptic buzz when a ‘slumped’ posture is detected. On the other hand, LumoBack is worn at the waist and encourages self-surveillance via a smartphone application but does not provide any haptic feedback. Users of all these devices will be able to track and monitor their daily posture with the accompanying smartphone application. Further details about these devices as well as other posture wearables will be discussed later in this paper. Takasaki demonstrated that the LumoBack is reliable at evaluating pelvic posture and can differentiate between healthy individuals and those with chronic LBP [21]. However, the lack of significant quantitative validation for these devices may suggest that Takasaki’s trial only demonstrates the potential of commercial devices to assess postural changes.

### Biomechanical Studies in Postural Analysis

Acclerometry devices such as the activPAL, Intelligent Device for Energy Expenditure and Activity (IDEEA) and DynaPort MoveMonitor have demonstrated the ability to discriminate posture [22]. However, these wearable sensors are mostly used in the research field to measure normal posture, as opposed to a real-world setting.

A study in 2016 demonstrated that tri-axial accelerometers can accurately differentiate static orientations from dynamic motions [22]. The comparison of accelerometry data to rater identification of various activities demonstrated median sensitivities of above 98% for static positions such sitting and lying down, and a sensitivity of 86% for standing. Median sensitivities for dynamic movements of walking and jogging were greater than 96%, whereas postural transition showed median sensitivity of 87% [22]. As smartphones often have accelerometers built in, Koumantakis et al. [23] studied the validity of a smartphone application for postural analysis. They demonstrated that built-in accelerometers in smartphones can measure sagittal low back and pelvic inclination with intracorrelation class coefficents (ICCs) of 0.96 and 0.97 for lumbar curve and sacral slope respectively [23]. Previous studies have also shown smartphone-based accelerometry data in posture analysis to be valid [24,25]. Although some studies were able to prove the validity of such devices, these studies were mostly carried out in a controlled environment with a small sample size and for a short duration. Furthermore, they also did not demonstrate the ability of accelerometers to delineate spinal pathologies from healthy individuals based on postural parameters.

Despite numerous studies on postural analysis, be they lab-based or using wearable devices, there is no gold standard for objective postural measurement, with no clear definition between good and poor posture due to a lack of standardized scoring algorithm for posture parameters.

## 4. Methodology—Device Market Review

**Figure 1:**
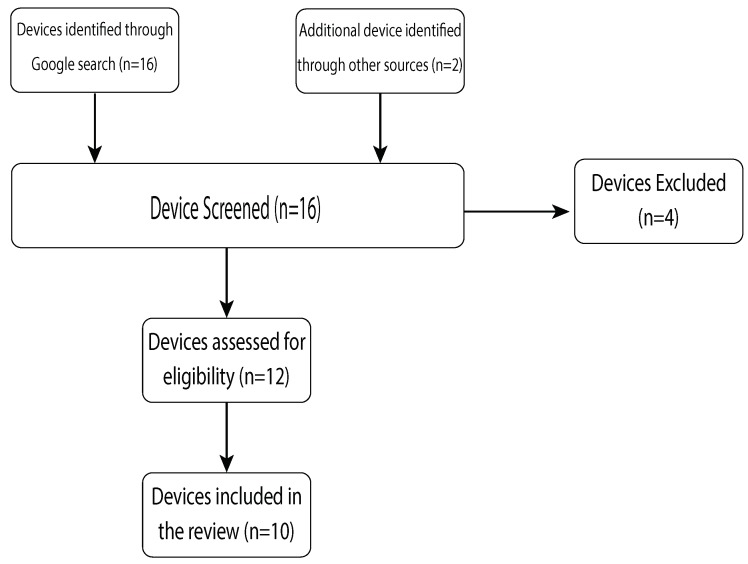
Flowchart of device search strategy.

A Google search was conducted in July 2019 to obtain information on posture wearable devices available on the commercial market. Search terms used were Posture, Wearable, device and Commercial. Inclusion criteria were wearable technologies and wearables capable of monitoring and recording posture. Only devices which are commercially available in Australia were included. Exclusion criteria included devices which corrected posture via mechanical means such as in a form of a brace. Products whose device specifications were unknown or unavailable to the authors were not eligible for the review. Search results were screened and are shown in Figure 1. A total of 10 commercially available posture devices were found and included in this study.

## 5. Commercially Available Postural Devices

With a greater focus on health, there has been a boom in the consumer market for wearable devices capable of measuring health parameters. This section aims to review wearable postural devices available on the commercial market. Details on how these devices determined posture and how the measurements were obtained were not available, as the devices were for commercial use, as opposed to research purposes. The authors approached the respective companies; however, no company was willing to divulge this information, citing proprietary right. Table 3 shows the comparison of product specifications among the postural devices discussed below.

### 5.1. Upright Go

Upright Go is a wearable posture trainer that is attached to the back via hypoallergenic adhesives which can be reused up to 10 times. The adhesives are made out of medical grade silicon to prevent skin irritation. This device aims to promote excellent long term posture habits via a coaching program on the accompanying app. Two modes are available; namely, training and tracking, with gentle vibrations provided if a slouched posture is detected in the former. A study is currently underway at Colombia University to investigate the use of Upright Go as a posture training tool. Upright Go 2, the newest version of Upright Go, has just been launched in 2019. The Go 2 differs from its previous model, with three times longer battery life, the use of multiple sensors as opposed to a single sensor and is 50% smaller and lighter than the original.

### 5.2. Lumo Back

LumoBack consists of a haptic motor, microcontroller, accelerometer, Bluetooth chip and flash memory. Its algorithm normalizes and calibrates accelerometer data to be converted into angular data. Attached via a belt, the device measures the angle of the pelvis when sitting or standing with a resolution of 1°. Preliminary study findings have revealed postural score differences between asymptomatic individuals and those with low back pain (LBP) using this device. The LumoBack device has also shown acceptable test-retest reliability for the posture score and time spent sitting [21].

### 5.3. Lumo Lift

From the same company that is behind Lumo Back, Lumo Lift is made up of the same basic components but differs in that it also has a gyroscope. As the device is designed to be accompanied by a smartphone application, a Wi-Fi chip inside enables postural data to be sent and viewed in the application [26]. Postural biofeedback is given to users to monitor and coach posture. Studies have demonstrated Lumo Lift as a practical lifestyle intervention to improve posture over time in asymptomatic individuals as well as reducing pre-existing LBP discomfort [27]. However, the detection of activity transition is limited, with the Lumo Lift unable to provide accurate postural feedback during lifting in daily life [28].

### 5.4. Zikto Walk

The Zikto Walk is made up of an accelerometer, gyroscope, memory, input unit, display unit, control unit, alarm unit and a communication module. Designed as a smart band, it measures the peak angle of arm swing to determine displacement, and hence, acceleration of the user. The motion data created is then stored and transformed into a motion score. The alarm unit serves to remind users when a lower than normal motion score is detected. Its multi-purpose design enables the device to measure parameters of health such as posture, step count and calories burnt. A multi-center prospective study is currently underway utilizing Zikto Walk as a physical activity tracker to prevent the onset of diabetes in high-risk individuals [29].

### 5.5. Prana

Unlike the Lumo and Upright Go devices, Prana is a breath training device which can measure respiration patterns and posture when positioned at the waist. Its circular disc-like design encompasses a microcontroller, and a memory and Bluetooth chip to send data to the accompanying smartphone application. It also contains two motion sensors; namely, an angle sensor and a displacement sensor. The angle sensor tracks posture angle, whereas the displacement sensor measures trunk circumference change. Posture position can be calculated based on the measured posture angle relative to a sampled range. On the other hand, trunk circumference change is relative to diaphragmatic movement. Thus, by further computing posture to the change in circumference with the baseline of complete inhalation and exhalation, the effect of posture on the movement of the diaphragm can be eliminated.

### 5.6. Jins Meme

Taking a novel approach to integrating wearable technology into healthcare, Jins Meme is an innovative eyewear that measures eye movements, as well as nose and eye positions, to determine levels of concentration, fatigue and posture. Three type of sensors are built into the frame of glasses: three-point electrooculography sensors, an accelerometer and a gyroscope. The electrooculography sensors are embedded in the nose pads to detect changes in blinking and eye movements. The accelerometer and gyroscope at the earpiece measure head rotational movements and posture. Jins Meme can be paired with a variety of smartphone applications, each designed for a specific purpose. For instance, JINS MEME DRIVE gives onscreen and audio cues to alert the user of possible drowsiness in order to ensure safe driving. JINS MEME application records ‘Mind Age’, the predicted mindfulness and productivity via detection of eye movement, and ‘Body Age’, the measure of physical activity, such as movement, posture and stability. Posture is calculated based on the balance of body axis perpendicular to the ground, whereas stability is the duration of maintaining such posture.

### 5.7. Alex+

Alex+ is a smart wearable that detects posture by measuring angles of the user’s neck. Designed to be worn around the neck and over the ears, it gives haptic feedback via vibrations when a ‘bad’ or ‘slouched’ posture is detected. Alex+ aims to develop good postural habits via posture tracking and individualized coaching program. Guided posture goals and exercise programs are provided via the accompanying app with the goal of breaking old habits of bad posture within 21 days.

### 5.8. Nadi X

Nadi X provides a unique twist to wearable technology as it is a pair of yoga pants with woven sensors capable of detecting body movements. It is used solely for practicing yoga, with built-in sensors at the hips, knees and ankles which vibrate to guide the user into correct yoga poses. Nadi X also features a device that powers such vibrations known as the Pulse. The Pulse is a three-axis accelerometer which attaches behind the user’s left knee and connects to a smartphone via Bluetooth. The Nadi X app provides step-by-step instructions on yoga poses with lesson difficulty ranging from beginner to guru.

### 5.9. Sense-U

Sense-U is designed to be clipped magnetically onto the user’s clothing and consists of an accelerometer, a gyroscope and a magnetometer; it measures body motions in nine DoF. It functions to detect daily energy expenditure, posture, sleep quality and dangerous events such as falling, which is potentially valuable for the elderly population. Motion data is sent via Bluetooth to an accompanying smartphone app that shows a visual representation of physical activity. For Apple smartphone users, Sense U also integrates with the Apple HealthKit which can further increase integration into the individual’s daily life.

## 6. Clinical Applicability

Wearable devices capable of measuring and analyzing spinal posture could prove promising in the clinical setting for preventing, monitoring and treating spinal conditions, as well as other musculoskeletal conditions.

### 6.1. Prevention of Spinal Conditions

The use of computers, smartphones and tablets in our daily life for work and leisure mean that we now spend many hours in poor posture as our heads and neck bend towards the screens of such devices. The human head weighs about 3–5 kg. But as the neck bends forwards and downwards, the effective weight on the cervical spine begins to increase. At a 15° angle, this weight is about 12 kg, at 30° it is 18 kg, at 45° 22 kg, and at 60°, it is 27 kg [30]. This epidemic known as ‘text neck’ and years of poor posture can lead to serious implications for our spinal health. Ergonomic strategies have been introduced in workplaces such as sit-to-stand desks in offices and manual handling training for those in the manual workforce. Despite such strategies to prevent occupational hazards, returning to a bad or unsafe posture is largely inevitable. Several reasons which could be attributed to this include individuals reverting to previous bad posture habits if not reinforced, emergency situations in manual tasks, for example, sudden movements; and stressful working environments which will not eliminate such risks [31]. Long periods of desk work accompanied by static posture, workplace design and working posture have been associated with the development of musculoskeletal neck and back issues. Monitoring and providing real-time biofeedback of seated spinal posture would prove valuable in preventing such disorders. The possibility of integrating clothing with a wearable plastic optic fiber was explored with optimistic outcomes. The study involving nine subjects demonstrated that the sensor could approximate the accuracy of expert visual analysis and provided sufficient measurement reliably when monitoring seated spinal posture [32].

Due to the nature of their jobs, construction workers often exceed their natural physical capabilities and undergo sustained physical labor. Work-related musculoskeletal disorders (WMSDs) of the lower back and neck are prevalent in this population and are precursors to operational injuries. Yan et al. proposed a real time wearable IMU motion warning system integrated into construction workers’ personal protection equipment to enable self-prevention of WMSDs without causing distractions during these operations [33]. Healthcare professionals believe that maintaining good posture plays a role in the prevention of spinal and musculoskeletal conditions. Nonetheless, there is insufficient statistical evidence to prove this relationship, as it would take years of observational studies and research in order to confirm such a link.

### 6.2. Monitoring of Spinal Conditions

As wearable posture devices are able to monitor and provide real time biofeedback on one’s posture, they can offer healthcare professionals a valuable insight into patients’ daily postures. A potential application of such a wearable is to be accompanied by two smartphone apps; namely, one for the patient and one for the physician. Patients would be able to view their posture history, such as hours of good posture per day, via graphics. Healthcare professionals would also be able to view that information via their app, and hence, monitor patients’ postures remotely. This would be particularly useful for monitoring patient progress pre and post-spinal intervention. Given time limitations of busy healthcare providers, there is a need to develop such technologies that can not only collect postural data, but provide adequate infrastructure and platforms that can relate this information in a simple manner for clinicians, rehabilitation providers or physical therapists. Integrating remote postural data into medical records to be treated as any other investigation modality could be a potential use for clinicians to be better able to utilize postural metrics in the decision-making process. Certain spinal pathologies affect posture as patients adopt an abnormal posture in order to adapt to changes in the spine or for pain alleviation. These pathologies include lumbar spinal stenosis (LSS), myelopathy, sciatica and low back pain (LBP) which are also ideal for monitoring with a wearable as the symptoms can be treated with both conservative and surgical approaches. Treatment response to both approaches can be determined via a wearable device which tracks patients’ postures in their daily lives during the pre and post-intervention stages. Besides posture, the use of wearable devices for gait analysis in LSS patients has received significant attention as evidence suggests these patients demonstrate detectable gait metrics differences compared to normal subjects [34]. The use of a commercial wearable device for detecting gait metrics in LSS is promising with a proposed objective scoring algorithm—the gait posture index (GPi), potentially providing an objective measure of patient function post-surgical interventions [35]. Furthermore, wearable devices can play a vital role in rehabilitation programs, as posture awareness is an essential component. Postural changes for instance, during standing, walking and sit-to-stand transitions are vital in accessing the efficacy of rehabilitation. However, direct visual observations of one’s posture or interviews to evaluate improvements are subject to both assessor and self-bias. Hence, there is a need for objective measurements of posture which can be conveniently carried out at rehabilitation centers and homes. A wearable posture device can address this gap in order to quantitatively evaluate postural changes during rehabilitation and activities of daily living.

### 6.3. Treating Spinal Conditions

Kyphosis is the sagittal curvature of the spine with a posterior convexity. This curvature in the sagittal plane is typically smooth with a range between 20° and 45° according to the Scoliosis Research Society classification system [36]. Postural hyperkyphosis is a common spinal curvature disorder [37] resulting from high external loads applied onto spines of individuals with poor muscle strength when in the upright position [38]. Hyperkyphosis is linked to rapid degeneration of the spinal column and disorders of the cervical and thoracic vertebrae [39]. In growing adolescents, hyperkyphosis can affect viscera development with respiratory function altered in severe thoracic hyperkyphosis. The evidence suggests that hyperkyphosis is linked to misalignment in the sagittal plane. Prolonged periods of sitting, as well as poor sitting and standing posture can lead to such sagittal misalignment in the adolescent population [40].

Orthopaedic and surgical treatments are the current mainstay approaches for postural kyphosis. The indication for orthopaedic methods is round kyphosis of less than 65° in an individual who has at least one year of remaining growth. This approach involves physiotherapy which includes corrective exercises and braces. The aim of physiotherapy is to strengthen trunk muscles and stretch the hamstrings which could aid in pain reduction. The role of corrective exercises is restoration of the spine to its normal position and movement, as well as maintenance of good posture during static and dynamic loads [41]. A posture wearable can be useful in physical therapy and corrective exercises by providing an objective measurement of posture, as mentioned in the section above. Ideally, the device could also provide real time haptic feedback so patients would be able to better adhere to such programs for better results.

### 6.4. Other Uses of Posture Wearables in Healthcare

#### 6.4.1. Falls Risk Assessment

Falls are a prevalent issue in the elderly population and can lead to serious physical and psychological consequences. As falls are multifactorial, devising appropriate strategies and interventions for fall prevention relies on fall risk assessments. Clinical fall risk assessments are traditionally comprised of questionnaires and functional assessments of gait, posture, cognition and other risk factors for falling. These assessments are subjective, qualitative and often oversimplify geriatric falls risk factors by binarily categorizing the elderly into fallers and non-fallers [42]. Typically, objective fall risk assessments are carried out in a laboratory with the use of complex motion sensor systems. This is usually complex and can only be set up in laboratories; hence, it is a time-consuming process to set up and integrate into a clinical setting. That being said, the use of wearables at home and in clinical settings have been well established for Parkinson’s disease [43,44,45,46]. Unfortunately, this application does not translate well into the treatment and monitoring of other pathologies involving postural changes such as lumbar spinal stenosis and myelopathy. Studies have shown certain gait and posture metrics to be associated with falls risk; however, the validity of these findings is uncertain. It is not guaranteed that characteristics indicating falls risk when measured in a laboratory will translate into falls in daily life. Wearables consisting of IMUs can address this gap by providing continuous quantitative measures of fall risks in daily life. In addition to fall detection, fall prediction is likely to be a more important metric. It is well-established in the literature that low gait velocity is correlated to high fall risk [47]; however, this relationship is not straightforward, with many other factors impacting fall events. Other metrics associated with falls risk will be discussed below.

**Figure 2:**
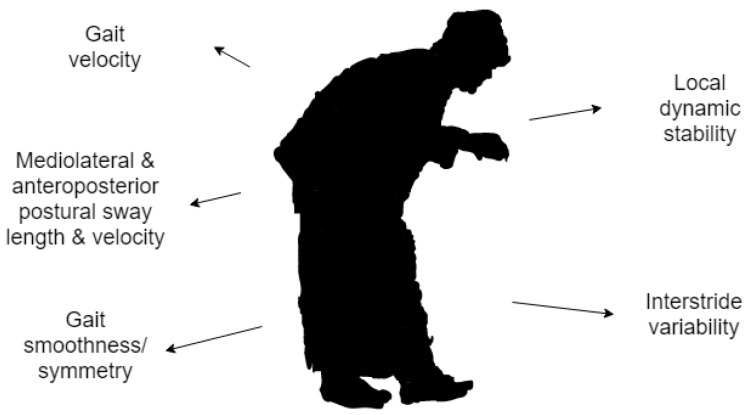
Gait and posture metrics in fall events [42,48].

A variable linked to falls risk is low gait velocity; that being said, slow gait may be an accommodation linked to fear of falling as opposed to an indicator of high fall risk. Mediolateral and anteroposterior postural sway length and velocity, which represent trunk movements during static standing, are measures used in the classification of fall risks. Large values of gait smoothness measured from the root mean square of linear acceleration are also associated with falls [42]. Furthermore, Rispens et al. have demonstrated that controlled laboratory measurements of local dynamic stability, gait velocity, interstride variability and gait symmetry are significantly associated with fall incidence. Their study shows that daily accelerometry data from wearables can reliably determine such gait characteristics in fall events [48]. Gait and posture metrics associated with fall risk are shown in Figure 2.

#### 6.4.2. Fall Detection

As posture wearables can detect body movements in several planes, they can serve as fall detection systems, especially in the geriatric population. Each year, approximately 30% of the elderly population in a developed country fall at least once, with 10–20% falling twice or more [49]. The global rise of athe ging population means that there has been an increase in healthcare demands, with a struggle to cope with the increasing needs of an aging population. Falls are critical events, particularly for the elderly as they can lead to increased morbidity, mortality and debilitating loss of quality of life. About half of those who remain on the ground for more than an hour after a fall die within 6 months, even without direct injury from the fall itself [50]. Therefore, timely rescue of the elderly is needed in order to avoid such devastating events. In this era of technology, telehealth applications such as wearables for unobtrusive monitoring of the aging population, especially community-dwelling elderly, would prove valuable. Telehealth is the provision of health services such as communications, information, measuring and monitoring remotely to the patients’ homes [51]. A wearable can automatically detect a fall, generate an alarm and send the location of the individual to the assigned emergency contact. Fall detection can be carried out by its accelerometer component by identifying typical changes in acceleration during a fall then analyzing these changes. Postural wearables come into play as they can recognize the difference in an individual’s orientation before and after a fall, and supine and prone positions after an impact [50].

### 6.5. The Use of Wearables in Other Diseases

#### Parkinson’s Disease

The cardinal features of Parkinson’s disease (PD) are tremor at rest, rigidity, akinesia or bradykinesia [52] and postural instability. PD patients also experience an array of postural and gait complications, including flexed posture, decreased postural responses, freezing and dysrrhythmic gait [52,53]. Postural instability and falls typically occur during later stages of PD and can be considered the most disabling motor symptoms. These symptoms are believed to be from a multitude of factors such as stooped posture, decreased postural reflexes and cognition, hypokinesia and diminished postural responses. Biofeedback has been implemented in healthcare to train and enable individuals to learn to modify their behavior. For instance, biofeedback training in posture and balance has proven effective in improving postural control among adolescent scoliosis patients. Similarly, this feedback loop can be applied for postural improvements among PD patients. A study in 2011 utilizing audio-biofeedback training for PD demonstrated that such training is feasible and is associated with quantitative improvements [53]. Nanhoe-Mahabier et al. [54] have shown vibrotactile biofeedback training in PD patients to be effective, while another study by Carpinella et al. [55] suggested biofeedback training to be superior to traditional methods of physiotherapy. Therefore, if this approach could be integrated into a posture device, this could be the first step in establishing a home-based training and monitoring program, as opposed to the traditional settings of physiotherapy clinic or rehabilitation centers for PD patients. Healthcare professionals would also be able to monitor disease progression and assess the effectiveness of treatment modalities via objective measurements of posture.

### 6.6. Multiple Sclerosis

Multiple sclerosis (MS) is a chronic progressive central nervous system (CNS) disorder with various neurological functions affected, including gait, balance, muscle tone and strength, cognition, vision and sensation. Loss of balance is one of the initial symptoms of MS which could lead to increased falls risk among MS patients. Studies have shown that individuals with MS have three related abnormalities of balance control. Not only is their ability to maintain a position is decreased, they have delayed responses to postural perturbations and change. These patients also have slow and limited movements towards their limits of stability [56]. In a study by Sun et al. [57], a wearable inertial sensor system demonstrated excellent correlation in postural sway metrics among MS patients in comparison to the gold standard of using a force plate and externally validated inertial sensor. This illustration of pelvic sway might be the key in understanding mobility impairments in MS. People with MS also have dynamic sway patterns not present in the healthy population. A study which incorporated gyroscopic corrections in accelerometry data was able to recognize asymmetric pelvic sway in MS patients when walking [58]. Hence, posture wearables have the potential to be a valid measurement tool not only in assessing and understanding disease progression, but also in early identification of MS patients.

### 6.7. Stroke

Stroke is one of the leading causes of long-term disability in Western countries, particularly in the United States where approximately 700,000 people are affected by stroke each year. Many stroke patients suffer from motor dysfunction, such as mobility impairments related to gait and difficulty in postural transitions and loss of balance [59]. Stroke recovery is one which extends beyond hospital wards, into rehabilitation programs and home recovery. Post-stroke rehabilitation requires regular physiotherapy and occupational therapy sessions which are guided by clinical assessments of motor abilities. Similar to those carried out for fall risk, these assessment are subjective and subject to potential bias. These therapy sessions can also be costly and require frequent visits to rehabilitation clinics. Thus, there is a role for postural wearables to provide objective clinical assessment scores, and be used as a rehabilitation tool. The wearable can also be used at home and at rehabilitation centers to accurately guide the rehabilitation process. Remote monitoring of the patients at home can be performed by the healthcare provider to track patients’ recoveries. The ease of a wearable which could be used in a home setting for rehabilitation programs will not only empower patients, but the therapists as well.

Stroke survivors can suffer from ‘pusher syndrome’, which is a clinical disorder following left or right brain injury in which patients actively push away from the non-hemiparetic side, resulting in loss of postural balance [60]. A study by Arteaga et al. proposed an accelerometry posture monitoring system for these patients whereby audio feedback was provided when poor posture was detected to alert and allow for self correction in free-living scenarios [61]. This avenue has been well explored, as regaining the ability to walk after a stroke is a major rehabilitation goal. Other wearable systems which have been proposed to assist stroke survivors include a ‘smart shoe’ capable of recognizing postures and physical activities [62], a sensorized garment to guide patients into correct rehabilitative exercises [63] and a wearable IMU to monitor therapeutic efficacy for diagnosing stroke in PD patients in clinical settings [64].

## 7. Discussion

### 7.1. Challenges and Future Steps in Wearable Technology

As the use of wearable technology in monitoring health parameters is still in its infancy, there are some obstacles in the integration of such devices into an individual’s daily lives.

Financial affordability is a factor that could affect user wearability. Most wearable devices are not cheap, with the most affordable device mentioned in the previous section costing approximately USD 100. The link between health and socioeconomic status has long been demonstrated in studies both in Australia and worldwide, with individuals from lower socioeconomic status at greater risk of poor health. Therefore, wearable devices for monitoring health seem to appeal to those who need them the least. A survey conducted among users of wearable devices showed that 49% were below the age of 35 and 29% reported an annual earning of more than USD 100,000 [65]. In order to better promote patient engagement with wearable technology, steps have to be taken in terms of increasing device affordability and accessibility. An example is for medical insurance companies to fund such wearable devices as part of the treatment regime as they would prescription medication.

Another challenge in using a wearable is the practicality of integrating it into daily life. There is typically no external reminder to wear such wearables, unlike the use of face masks, whereby one can receive an environmental stimuli as a reminder by observing the poor air quality [66,67]. Health-related behavioral change can only bring about improvements to health parameters if the changes can be sustained. Wearable users will have to remember to wear the device on a daily basis and charge it, requiring additional efforts from those who may not be able to sustain such a lifestyle. In a survey of more than 6000 wearable users, half reported having stopped using their device, with one third doing so before 6 months of use [68]. Therefore, a viable solution for this non-compliance is to build upon a previous behavior rather than creating a new habit and maintaining it. Utilizing a smartphone with an application capable of measuring health parameters such as posture could be a potential solution. Despite their price tag, a large portion of the general population own a smartphone which is carried on their person throughout the day. Hence, they would not need to spend additional money or time on a separate wearable device, since a smartphone would not require any effort beyond setting up an app. Regularly charging a phone is a task most are already accustomed to as well. Although there has been an increased interest in the use of wearable devices for healthcare, the accuracy of such devices is relatively unknown.

### 7.2. Posture Wearables and Telemedicine

The traditional model of care is paternalistic, with patients fully reliant on healthcare professionals to navigate the complexity of the healthcare ecosystem. Healthcare professionals retain more power as patients depend on them for information, diagnosis and treatment. The model of care now has started to shift towards one based on empowered patients sharing ownership, whereby patients can self-manage and assume personal responsibility for their health. The rise of consumer health werarables has enabled patients to take up a more active role in health maintenance.

The use of wearable devices is an exciting avenue which has been garnering interest in both the technology and healthcare industry. Wearables are rapidly gaining traction as they are small, lightweight and can be integrated easily into an individual’s daily life. They are typically paired with a smartphone application which allows the end-users to better engage and assume personal responsibility for their health. This enables the integration of wearables with smart devices which can lead to real-life postural analysis. Furthermore, constant postural monitoring would be able to give continuous and objective measurements, as opposed to a single-time solitary data collection in a laboratory. Certain wearables can even send real-life haptic biofeedback which offers additional motivation and feedback to improve compliance; hence, improving patients’ clinical outcomes [69]. These posture trainers provide historical postural tracking via visual graphics in the accompanying app which can further motivate users through a feedback loop. The objective nature of posture wearables eliminates the concern of subjective bias, making them an ideal candidate in the remote monitoring of patients in a clinical setting [70]. However, the lack of standardized objective measurements remains a hindrance for continuous monitoring as there is no definite delineation between good and bad posture. There is also insufficient research in postural data and for the depiction of spinal pathologies based on such postural metrics.

## 8. Conclusions

The current gold standard for postural analysis is via radiography, with photogrammetric methods emerging as an alternative. However, these lab-based approaches are not suitable for monitoring one’s posture on a day to day basis. In response, wearable technology could address this gap. Wearable devices are able to provide objective measurements of one’s posture. That being said, the lack of standardized delineation of posture remains a hindrance. Although there is a growing trend of commercial wearable devices consisting of accelerometers, gyroscopes and magnetometers for remote day to day data acquisition, significance studies need to be carried out to confirm their validity. Continuous data collection may allow for the detection of spinal conditions based on postural metrics to identify these patients quicker and easier. On the other hand, the line between smart health wearables and medical devices has started to blur with the advancements in technology. This has allowed patients to facilitate preventative care, contribute directly to their health and manage ongoing conditions. The use of commercial wearables for healthcare has its own pros and cons. Healthcare professionals might be overwhelmed with the increase of patients bringing wearables to consultations and self-diagnosing based on that data. This can then lead to confusion and tension between health practitioners and patients. Alternatively, healthcare professionals and researchers could work hand in hand in to validate wearables as a supporting tool which would then be incorporated into the healthcare system [7]. In conclusion, the application of this wearable technology in healthcare is in its infancy, and how it may best serve medicine remains unclear.

## Figures and Tables

**Table 1:** Components of inertial measurement units (IMUs) [4].

Components	Function
Accelerometer	Measure proper acceleration:
	— Gravitational force (static) and sensor movement (dynamic)
	— At least 1-D accelerometer
Gyroscope	Measure angular velocity:
	— At least 1-D gyroscope
Magnetometer	Measure all magnetic fields:
	— Optional

**Table 2:** Degrees of freedom (DoF) in IMUs [4].

DoF	IMU Components
9	3-D accelerometer, 3-D gyroscope & 3-D magnetometer:
	— Most accurate type → able to measure proper acceleration, angular
	velocity and magnetic fields in three axes
6	3-D accelerometer & 3-D gyroscope:
	— Less accurate than 9 DoF → lower accuracy in determining sensor orientation
5	3-D accelerometer & 2-D gyroscope:
	— Less accurate than 6 DoF → gyroscope cannot measure in third dimension
4	3-D accelerometer & 1-D gyroscope:
	— Less accurate than 5 DoF → gyroscope can only measure in one dimension

**Table 3:** Specifications of commercial posture wearables.

	Upright Go	Upright Go 2	LumoLift	LumoBack	Alex	Nadi X	Sense-U	Zikto Walk	Prana	Jins Meme
Size (mm)	55.3 × 33.2	48 × 28	44 × 25	415 × 100	80 × 160	NA	35.6 × 35.6	13.6 × 47.3	31.8 × 6.4	NA
(Length × Width × Height)	×11.6	×8.6	×13	× 8	×170		×10.2	×11.1	(Height × diameter)	
Weight (g)	12	11	13.6	25	25	NA	11.34	17.5	I.N.A.	36
Accelerometer (acc.)	1	1	1	1	1	1	1	1	2	1
Type of acc.	NA	NA	MEMS	NA	MEMS	MEMS	NA	NA	I.N.A.	MEMS
No. of acc. axis	3	NA	3	3	NA	3	NA	3	3	3
Gyroscope (gyro.)	NA	1	NA	NA	NA	NA	NA	1	I.N.A.	1
Type of gyro.	NA	NA	NA	NA	NA	NA	NA	NA	I.N.A.	MEMS
No. of gyro. axis	–	–	–	–	–	–	–	3	–	3
Magnetometer (mgm.)	NA	NA	NA	NA	NA	NA	NA	NA	I.N.A.	1
Type of mgm.	NA	NA	NA	NA	NA	NA	NA	NA	I.N.A.	I.N.A.
No. of mgm. axis	–	–	–	–	–	–	–	I.N.A.	–	3
Sensor location	Upper back	Upper back	Clavicle	Waist	Neck	Hips, knees, ankles	Clavicle	Wrist	Waist	Nose bridge, ears
Battery type	Lithium (Li) ion	Li polymer	Li polymer	Li polymer	Li	Li ion	Li	Li polymer	Li ion	Li ion
Battery life (hours)	12	30	96	120–168	168	1.5	240	72–120	168	16

NA = Not applicable; I.N.A.= Information not available.
